# *Leishmania donovani* development in *Phlebotomus argentipes*: comparison of promastigote- and amastigote-initiated infections

**DOI:** 10.1017/S0031182016002067

**Published:** 2016-11-23

**Authors:** JOVANA SADLOVA, JITKA MYSKOVA, TEREZA LESTINOVA, JAN VOTYPKA, MATTHEW YEO, PETR VOLF

**Affiliations:** 1 Department of Parasitology, Faculty of Science, Charles University, Vinicna 7, 128 44 Prague 2, Czech Republic; 2 Department of Pathogen Molecular Biology, Faculty of Infectious and Tropical Diseases, London School of Hygiene and Tropical Medicine, Keppel Street, WC1E 7HTLondon, UK

**Keywords:** *Leishmania donovani*, *Phlebotomus*, promastigotes, amastigotes, transmission

## Abstract

*Leishmania* parasites alternate in their life cycle between promastigote stages that develop in the gut of phlebotomine sand flies and amastigotes residing inside phagocytic cells of vertebrate hosts. For experimental infections of sand flies, promastigotes are frequently used as this way of infection is technically easier although ingestion of promastigotes by sand flies is unnatural. Here we aimed to answer a critical question, to what extent do promastigote-initiated experimental infections differ from those initiated with intracellular amastigotes. We performed side-by-side comparison of *Leishmania* development in *Phlebotomus argentipes* females infected alternatively with promastigotes from log-phase cultures or amastigotes grown *ex vivo* in macrophages. Early stage infections showed substantial differences in parasite load and representation of morphological forms. The differences disappeared along the maturation of infections; both groups developed heavy late-stage infections with colonization of the stomodeal valve, uniform representation of infective metacyclics and equal efficiency of transmission. The results showed that studies focusing on early phase of *Leishmania* development in sand flies should be initiated with intracellular amastigotes. However, the use of promastigote stages for sand fly infections does not alter significantly the final outcome of *Leishmania donovani* development in *P. argentipes* and their transmissibility to the vertebrate host.

## INTRODUCTION

*Leishmania* parasites (Kinetoplastida: Trypanosomatidae), causative agents of leishmaniases, alternate in their life cycle between intracellular amastigote stages in vertebrate hosts and extracellular promastigote forms in phlebotomine sand flies (Diptera: Psychodidae). In sand flies, *Leishmania* undergo a complex development through several morphologically and functionally distinct forms (Bates, 2007; Dostalova and Volf, 2012). Sand fly females ingest blood containing macrophages infected with *Leishmania* amastigotes. In the abdominal midgut, the bloodmeal in engorged flies is encapsulated within the peritrophic matrix, a chitinous envelope secreted by midgut epithelial cells (Lehane, 1997). In this endoperitrophic space, amastigotes transform into procyclic promastigotes that multiply within the bloodmeal. During degeneration of the peritrophic matrix, procyclic promastigotes differentiate into elongated nectomonads, which escape into the ectoperitrophic space (Sadlova and Volf, 2009). Nectomonads first attach to the midgut epithelium, preventing excretion via defecation by the sand fly, later moving anteriorly to the thoracic midgut. Here they give rise to leptomonads which multiply and produce promastigote secretory gel, which fills the thoracic midgut and plays significant role in parasite transmission (Stierhof *et al.* 1999; Rogers *et al.* 2002). Finally, leptomonads either attach to the chitin layer of the stomodeal valve and differentiate into haptomonads or transform into small, highly motile infective metacyclics adapted for transmission to the next mammalian host (Sacks and Perkins, 1985; Bates, 2007).

Under laboratory conditions, either *Leishmania* amastigotes or promastigotes can be used for the initiation of experimental infection in sand flies. While promastigotes can be simply cultivated *in vitro* and infection is fast and uncomplicated, ingestion of promastigote forms by sand fly females is unnatural. In contrast, amastigote-initiated infections are more natural, but technically difficult and associated with ethical and safety concerns, which make their usage unfavourable. Assessing amastigote dose by direct feeding on infected hosts is difficult and those derived from organs of infected animals requires frequent animal sacrifice and are unavoidably contaminated with host material. Despite cultivation of axenic amastigote-like forms being relatively easy for some *Leishmania* species (Bates, 1993; Gupta *et al.* 2001), a large number of studies indicate considerable differences when comparing axenic amastigotes with intracellular ones on both genomic and proteomic levels (Holzer *et al.* 2006; Rochette *et al.* 2009; Pescher *et al.* 2011). For these reasons, the cultivation of amastigotes inside macrophages or macrophage-like cell lines is considered the best choice although it is relatively laborious and time consuming (Chang, 1980).

In our study, we aimed to answer the crucial question; to what extent promastigote-initiated experimental infections in sand flies differ from those initiated with amastigotes? If the pattern of development does not differ significantly then infection via promastigotes would be legitimate. Direct comparisons between these two methods are scarce and effects of this factor on transmission potential have not been compared. There is only a single similar study on *Leishmania infantum–Lutzomyia longipalpis* parasite–vector model (Freitas *et al.* 2012) which was, however, performed using axenic amastigotes that may substantially differ from intracellular ones due to the loss of important factors during a long-term *in vitro* culture (Pescher *et al.* 2011). Here we compared *Leishmania donovani* infection in its natural vector *Phlebotomus argentipes* using culture form promastigotes and amastigotes grown *ex vivo* in bone marrow-derived macrophages (BMM). We published previously (Pruzinova *et al.* 2015) that the Ethiopian *L. donovani* strain (GR374) used in the present study develops similarly in both African and Indian vector species – *Phlebotomus orientalis* and *Phlebotomus argentipes*, respectively. Additionally, for the first time, the transmission potential of parasites to the vertebrate host was assessed which is the most significant marker of successful *Leishmania* development in the vector.

## MATERIALS AND METHODS

### Sand flies and *Leishmania*

A laboratory colony of *P. argentipes* (originating from India) was maintained in the insectary of the Charles University in Prague under standard conditions (at 26 °C fed on 50% sucrose with a 14 h light/10 h dark photoperiod) as described previously (Volf and Volfova, 2011).

Amastigote *Leishmania* stages were grown in BMMs differentiated from precursor cells of BALB/c mice in the presence of L929 fibroblast cell culture supernatant as a source of macrophage-colony stimulating factor (M-CSF). *Leishmania donovani* (MHOM/ET/2010/GR374) promastigotes transfected with green fluorescence protein as described in Sadlova *et al.* (2011) were cultured in M199 medium (Sigma) containing 10% heat-inactivated fetal calf serum (Gibson) supplemented with 1% BME vitamins (Basal Medium Eagle, Sigma), 2% sterile urine, 250 *µ*g mL^−1^ amikacin (Amikin, Bristol-Myers Squibb) and 150 *µ*g mL^−1^ selective antibiotic G 418 (Sigma). Macrophages were exposed to stationary-phase parasites at a parasite-to-macrophage ratio of eight promastigotes to one macrophage. Both infected and uninfected macrophages were cultured in complete RPMI-1640 medium (Sigma) containing 10% FBS (fetal bovine serum), 20% L929 cell culture supernatant, 1% penicillin–streptomycin (Sigma), 2 mm of L-glutamine (Sigma) and 0·05 mm of *β*-mercapto-ethanol at 37 °C with 5% CO_2._

### Sand fly infections

To obtain amastigote stages, *L. donovani* were co-cultivated with BMM for 72 h and non-internalized parasites were removed by washing 3–5 times with preheated culture medium. Numbers of amastigotes per macrophages were counted by fluorescent microscopy of live macrophages. The infected macrophages were removed from the culture plates using trypsin–EDTA solution (Sigma), centrifuged at 300 ***g*** for 10 min and resuspended in heat-inactivated rabbit blood for sand fly infections at the concentration of 10^6^ amastigotes mL^−1^.

For promastigote-initiated infections, promastigotes from log-phase cultures (day 3–4 post-inoculation) were resuspended in heat-inactivated rabbit blood at concentration of 10^6^ promastigotes mL^−1^.

Sand fly females (5–9 days old) were pooled and infected by feeding through a chick-skin membrane either on amastigote- or on promastigote-containing suspension. Engorged sand flies were maintained under standard conditions. Females were dissected at days 1, 2, 4 and 8 post bloodmeal (PBM), and the abundance and location of *Leishmania* infections in the sand fly digestive tract was examined by fluorescent microscopy. Parasite loads were graded as light (<100 parasites per gut), moderate (100–500 parasites per gut) and heavy (>500 parasites per gut) based on Myskova *et al.* (2008). Experiments were performed in duplicate.

### Transmission by bite and collection of samples for quantitative real-time PCR (qPCR)

BALB/c mice were offered to experimentally infected *P. argentipes* at various time intervals PBM (from days 7–10 PBM). Sand flies (usually 10 females) were placed in a cage (20 × 20 cm^2^) and allowed to feed on the single mouse anaesthetized with ketamin/xylazin (150 mg and 15 mg kg^−1^, respectively) for approximately 1 h. Location of biting of each feeding female was recorded by drawing into the schematic picture of mouse body and engorged flies were collected by an aspirator immediately after terminating their bloodmeal. After exposure, mice were sacrificed by injecting them an overdose of ketamin/xylazin and localized tissue excised at the bite location. For sand flies which moved during the feeding, all feeding places were collected into one pooled sample. All samples (skin biopsies and corresponding fed sand flies) were stored at −20 °C until DNA extraction for qPCR.

### qPCR

Extraction of total DNA from rodent tissues and sand flies was performed using a DNA tissue isolation kit (Roche Diagnostics, Indianapolis, IN) according to the manufacturer's instructions. The qPCR for detection and quantification of *Leishmania* parasites was performed in Bio-Rad iCycler & iQ Real-Time PCR Systems using the SYBR Green detection method (iQ SYBR Green Supermix, Bio-Rad, Hercules, CA) as described previously (Myskova *et al.* 2008) using the kinetoplast primers (forward primer 5′-CTTTTCTGGTCCTCCGGGTAGG-3′ and reverse primer 5′-CCACCCGGCCCTATTTTACACCAA-3′) (Mary *et al.* 2004).

### Morphometry of parasites

**Fig. 1.**
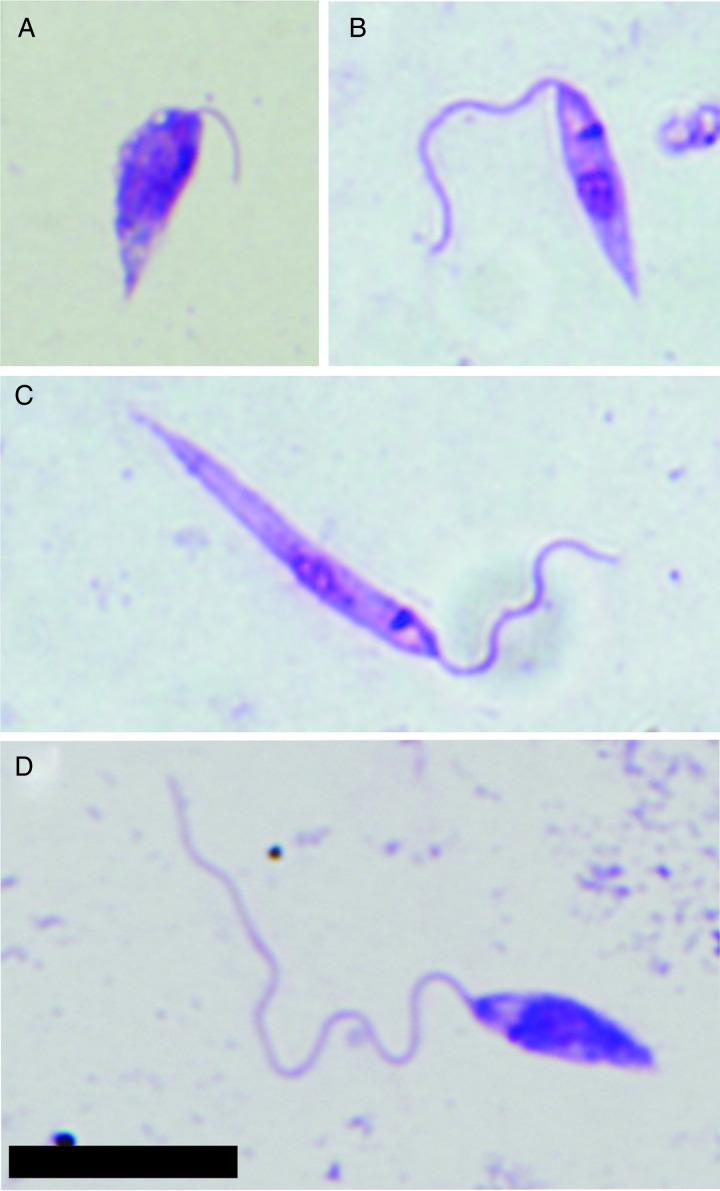
Morphological forms of *L. donovani* distinguished in this study based on criteria described in the Materials and Methods section. Parasites from midgut smears of infected sand flies were fixed with methanol and stained with Giemsa. (A) Procyclic promastigote; (B) short promastigote; (C) elongated nectomonad; (D) metacyclic promastigote. Scale bar = 10 *µ*m.

Midgut smears of sand flies infected with *L. donovani* were fixed with methanol, stained with Giemsa, examined by light microscopy with an oil immersion objective and photographed (Olympus DP70). Body length, flagellar length and body width of 120 randomly selected promastigotes from four females/smears were measured for each time-point PBM and both modes of infection using Image-J software. Four morphological forms were distinguished (Fig. 1), based on the criteria of Sadlova *et al.* (2010) and Rogers *et al.* (2002): (a) procyclic promastigotes, PP, flagellum < body length and body length < 14 *µ*m, present before defecation; (b) elongated nectomonads, EN, body length ⩾14 *µ*m; (c) metacyclic promastigotes, MP, flagellar length >2 times body length and body length < 14 *µ*m, present post defecation, and (d) short promastigotes, SP, body length <14 *µ*m and flagellar length ⩽2 times body length. The term ‘short promastigotes’ is derived from the terminology of Walters (1993) (short nectomonad promastigotes), which is the older synonym of leptomonads (leptomonad promastigotes) proposed by Rogers *et al.* (2002). Haptomonads were not distinguished in this study as they may remain attached to the gut and can be underrepresented on gut smears.

### Statistical analysis

Differences in intensities of infections, percentage of morphological forms found in infected flies and transmission efficiency were applied to Chi-square (*χ*^2^) tests. Differences between parasite loads, in sand flies and mice tissues respectively, were tested by non-parametric Mann–Whitney test. All statistical analysis was performed with the statistical software package SPSS version 23.

### Animal experimentation guidelines

Animals were maintained and handled in the animal facility of Charles University in Prague in accordance with institutional guidelines and Czech legislation (Act No. 246/1992 and 359/2012 coll. on Protection of Animals against Cruelty in present statutes at large), which complies with all relevant European Union and international guidelines for experimental animals. Female BALB/c mice were housed in standard plastic T3 cages (Velaz) in groups of six animals with *ad libitum* access to water and complete feed mixture ST-1 (Velaz). All the experiments were approved by the Committee on the Ethics of Laboratory Experiments of the Charles University in Prague and were performed under permissions no. MSMT-31114/2013-13 and MSMT-10270/2015-6 of the Ministry of the Environment of the Czech Republic. Investigators are certificated for experimentation with animals by the Ministry of Agriculture of the Czech Republic. Minimum numbers of animals to produce statistically reproducible results were used.

## RESULTS

### Development of parasites in *P. argentipes* females

**Fig. 2.**
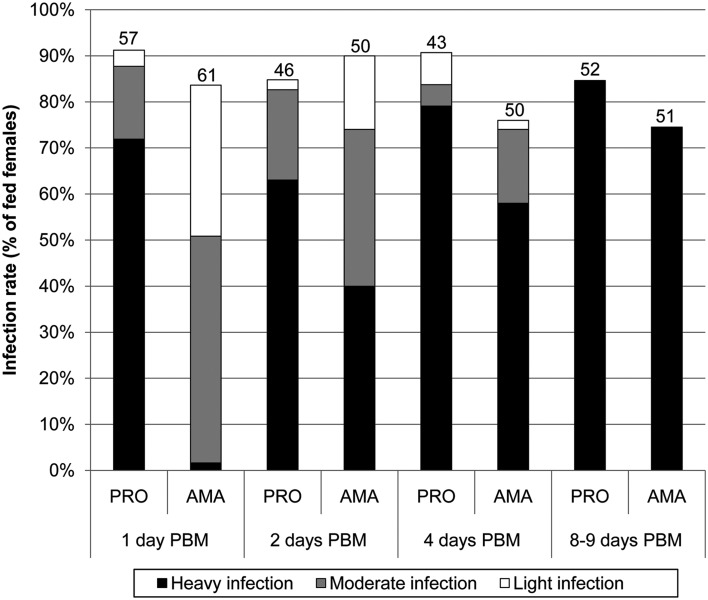
Infection rates (percent of females infected) and intensities of *L. donovani* infections in *P. argentipes*. Parasite load was estimated by fluorescent microscopy: light infections, <100 parasites per gut; moderate infections, 100–500 parasites per gut; heavy infections, >500 parasites per gut. Numbers of dissected females are shown above bars. PRO, promastigote-initiated infections; AMA, amastigote-initiated infections; PBM, post bloodmeal. Differences between groups were evaluated using Chi-square test: day 1 PBM, *P* < 0·0001, *χ*^2^ = 65·737, d.f. = 3; day 2 PBM, *P* = 0·021, *χ*^2^ = 9·743, d.f. = 3; day 4 PBM, *P* = 0·036, *χ*^2^ = 8·518, d.f. = 3; days 8–9 PBM, *P* = 0·203, *χ*^2^ = 1·6202, d.f. = 1.

During the first four days PBM, infections initiated with amastigote forms of *L. donovani* showed significantly lower intensity than promastigote-initiated ones (Fig. 2). The difference was most pronounced in very early infection, while infections initiated with promastigotes progressed quickly (72% of females showed heavy infection by 24 h PBM), ingested amastigotes underwent a substantial reduction in numbers before parasite load increased (only 2% of females showed heavy infections at 24 h PBM).

**Fig. 3.**
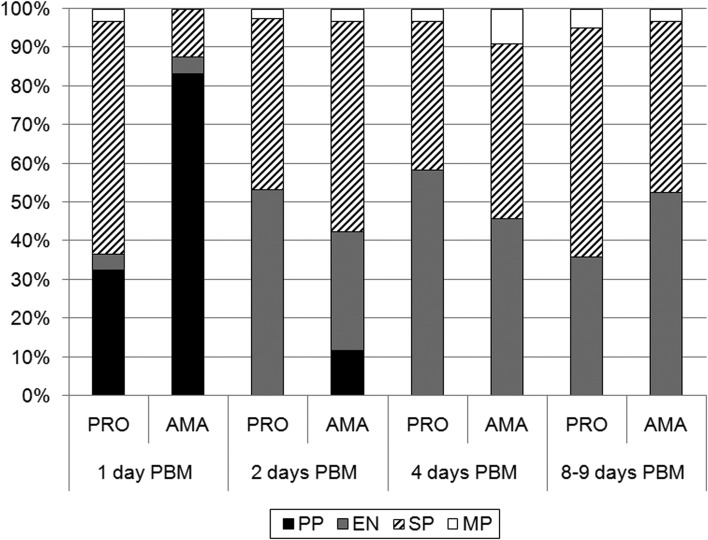
Morphological forms of *L. donovani* during development in *P. argentipes*. The guts of infected females were sampled at 1, 2, 4 and 8–9 days PBM and parasite morphometry was determined as described in methods. The percentage of each form found in infected flies at each time point is shown. PRO, promastigote-initiated infections; AMA, amastigote-initiated infections; PBM, post bloodmeal; PP, procyclic promastigotes; EN, elongated nectomonads; SP, short promastigotes; MP, metacyclic promastigotes. Differences between groups were tested by Chi-square test: day 1 PBM, *P* < 0·0001, *χ*^2^ = 68·115, d.f. = 3, day 2 PBM, *P* < 0·0001, *χ*^2^ = 22·581, d.f. = 3, day 4 PBM, *P* = 0·058, *χ*^2^ = 5·707, d.f. = 2, day 8–9 PBM, *P* = 0. 034, *χ*^2^ = 6·786, d.f. = 2.

Morphological differences between parasites in both groups were also most significant at 24 h PBM: procyclic promastigotes were most abundant in amastigote-initiated infections while short promastigotes dominated in promastigote-initiated infections (Fig. 3). By day 2 PBM, increased prevalence of elongated nectomonads in both groups was observed while procyclic forms were still present only in amastigote-initiated infections. Following sand fly defecation by day 4 PBM no substantial morphological differences were detected (Fig. 3).

**Fig. 4.**
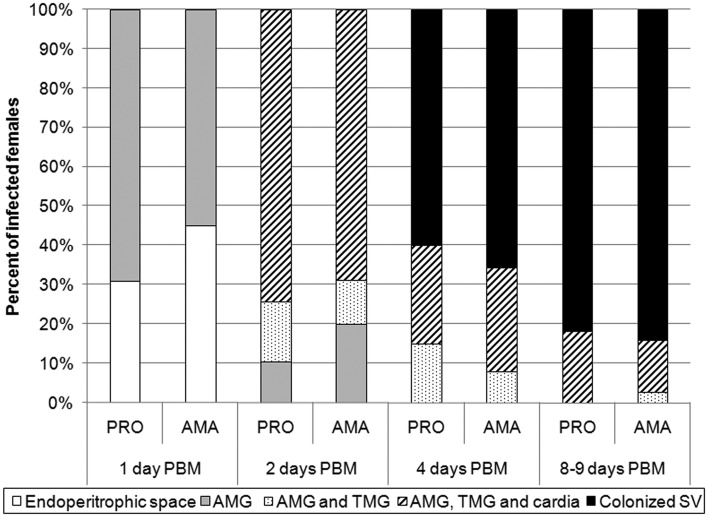
Location of *L. donovani* in infected *P. argentipes*. AMG, abdominal midgut; TMG, thoracic midgut; SV, stomodeal valve. Differences between groups were evaluated using *χ*^2^ test: day 1 PBM, *P* = 0·134, *χ*^2^ = 2·247, d.f. = 1; day 2 PBM, *P* = 0·436, *χ*^2^ = 1·661, d.f. = 2; day 4 PBM, *P* = 0·616, *χ*^2^ = 0·970, d.f. = 2; day 8–9 PBM, *P* = 0·473, *χ*^2^ = 1·497, d.f. = 2.

**Fig. 5.**
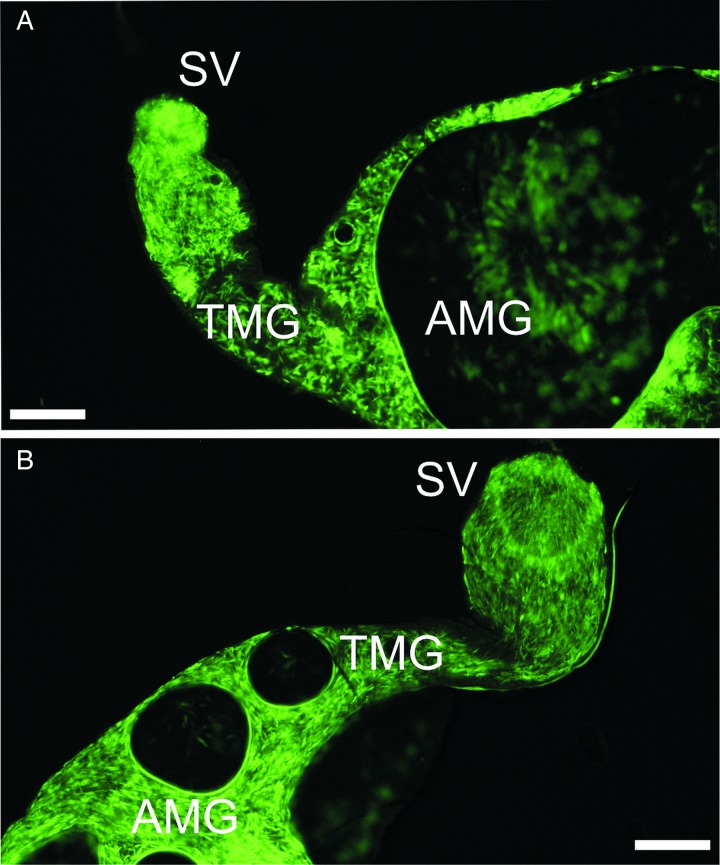
Images from fluorescent microscope showing amastigote-initiated (A) and promastigote-initiated (B) *L. donovani* infections in *P. argentipes* females by day 4 PBM. Parasites (transfected with GFP) are present in both abdominal and thoracic parts of the midgut and the stomodeal valve is colonized. AMG, abdominal midgut; TMG, thoracic midgut; SV, stomodeal valve. Scale bar = 100 *µ*m.

Interestingly, localization of infections did not differ significantly. In both groups, leishmania development was rapid; parasites escaped from the endoperitrophic space within 24 h PBM, by 48 h PBM parasites had reached the thoracic midgut, including cardia region and by day 4 PBM colonized the stomodeal valve in more than 60% of sand flies in both groups (Figs 4 and 5).

Late-stage development of parasites showed only minor differences between amastigote- and promastigote-initiated infections. All mature infections (by days 8–9 PBM) had heavy parasite load, including colonization of the stomodeal valve in more than 80% of sand flies in both groups (Figs 2, 4). Elongated nectomonads prevailed in amastigote-initiated infections, but short promastigotes in promastigote-initiated infections, however differences in presence of metacyclic promastigotes between the groups were insignificant (Fig. 3; *P* = 0·518, *χ*^2^ = 0·417, d.f. = 1).

### Transmission by bite on BALB/c mice

The qPCR proved that in both sand fly groups, more than 70% of females allowed a second feed on mice were infected with *Leishmania* parasites (Table 1) and that parasite loads in sand flies did not differ significantly between the groups (*N* = 117, Mann–Whitney *U* = 1600·0, *Z* = −0·369, *P* = 0·712). From *Leishmania*-positive flies, 42 and 56% delivered parasites into the skin of the mice in promastigote- and amastigote-initiated infections, respectively. This apparent difference in transmission efficiency is not statistically significant (*P* = 0·270, *χ*^2^ = 1·750, d.f. = 1). Numbers of parasites detected in mice tissues by qPCR were slightly higher in the group where sand flies were infected with amastigotes, but not statistically significant (Mann–Whitney *U* = 138·0, *Z* = −1·695, *P* = 0·090).

**Table 1.** Transmission efficiency of *Leishmania donovani* on BALB/c mice using *Phlebotomus argentipes* females

Mode of sand fly infections	Numbers of *P. argentipes* females			Parasite load in sand flies	Transmitted parasites
	Feeding on mice	*L. donovani* infected	Transmitting *L. donovani* (transmission efficiency)	Mean ± s.d. (median)	Mean ± s.d. (min, max)
Promastigote-initiated	49	36	15 (42%)	68 090 ± 8 628 (59 500)	14 ± 3 (1–33)
Amastigote-initiated	68	48	27 (56%)	107 260 ± 15 672 (79 100)	58 ± 21 (3–530)

Parasite numbers were detected using qPCR.

## DISCUSSION

The results show that mature infections of *L. donovani* in *P. argentipes* initiated with promastigotes did not differ significantly from amastigote initiated infections in the context of parasite load, colonization of the stomodeal valve, presence of infective metacyclic stages and transmission efficiency. In both the compared groups, the development of *L. donovani* was rapid; parasites escaped from the endoperitrophic space before 24 h PBM and reached the cardia by day 2 PBM with colonization of the stomodeal valve by day 4 PBM. This rapid progress is typical for *P. argentipes* and is caused by very fast bloodmeal digestion followed by degradation of the peritrophic matrix and defecation of blood remnants by day 3 PBM (Pruzinova *et al.* 2015). In contrast there was a pronounced difference between promastigote- and amastigote-initiated infections observed during the first days PBM in both morphology of parasites and kinetics of population growth. Therefore, the use of promastigotes is not recommended for studies on early-stage *Leishmania* development in sand flies.

As expected, both experimental groups differed in the context of morphological forms in early-stage sand fly infections. While procyclic promastigotes prevailed in amastigote-initiated infection, short promastigotes were predominant in promastigote-initiated infection. However, elongated nectomonads appeared at the same rate in both groups. Interestingly, localization of infections in sand fly midguts did not differ between the groups, as for example in the rate of parasite escape from the peritrophic matrix into the ectoperitrophic space of the abdominal midgut. The fact that the peritrophic matrix was degraded at the same time in both groups, independently of the prevailing parasite forms present, confirms previous findings on *Leishmania major*–*Phlebotomus duboscqi* interactions where a ‘sit and wait strategy’ was proposed for *Leishmania* parasites in natural vectors (Sadlova and Volf, 2009) and supports the idea that this process is mediated by sand-fly derived chitinases (Ramalho-Ortigão and Traub-Csekö, 2003; Ramalho-Ortigão *et al.* 2005) and not substantially influenced by the parasite itself.

In amastigote-initiated infection, substantial reduction of parasite numbers was initially observed, followed by recovery and number increase. The average blood volume ingested by *P. argentipes* females feeding through a chick skin membrane on rabbit blood was 0·6 *µ*L (Pruzinova *et al.* 2015), representing an initial inoculum of about 600 parasites. However, initial numbers of parasites in amastigote-initiated infections were <500 in 98% of females falling to <100 in 39% of females by 24 h PBM (>80% reduction). The early reduction of parasite numbers in infections initiated with lesion-derived amastigotes has been repeatedly described in *Leishmania mexicana*-infected *L. longipalpis* (reduction to 88 and 66% by 6 and 24 h PBM, respectively (Rogers *et al.* 2002) or reduction to 98–99% during the first 24 h PBM (Rogers *et al.* 2008) and in *L. major*-infected *P. papatasi* [almost 50% reduction by 4 h PBM (Pimenta *et al.* 1997)]. This loss of parasites has been attributed to early attack by digestive proteases in the sand fly gut (Borovsky and Schlein, 1987; Schlein and Jacobson, 1998).

The course of the experimental sand fly infections is influenced with the amount of parasites in the initial infective dose. Although in natural vectors even 1–2 parasites are sufficient for successful establishment of infection (Seblova *et al.* 2013; Pruzinova *et al.* 2015) higher infective doses, which are commonly used, are more efficient in producing higher infection rates in sand flies (Anjili *et al.* 2006; Seblova *et al.* 2013; Pruzinova *et al.* 2015). Higher initial doses of *L. major* promastigotes are known to increase parasite loads in *P. duboscqi* and result in a higher percentage of metacyclic forms and increased transmission frequency with more severe pathology in mice (Maia *et al.* 2011).

Stamper *et al.* (2011) showed that percentage of metacyclics in sand flies is one of the most important parameters predicting successful *Leishmania* transmission to the host. In the same study, authors did not find difference in frequency of metacyclics between *L. major* promastigote- and amastigote-infected *P. duboscqi*. Accordingly, our experiments with *L. donovani* showed similar numbers of metacyclic forms in mature infections as well as comparable transmission efficiency in amastigote- and promastigote-initiated *P. argentipes* infections. Different results were reported with late stage infections of *L. infantum chagasi* in *L. longipalpis* (Freitas *et al.* 2012). Here metacyclics were 10 times less represented in amastigote-initiated infections compare to promastigote-initiated infection. However, in this case axenic amastigotes were used and efficiency of transmissions on the host was not evaluated. Axenic amastigotes are not adequate alternative to intracellular amastigotes (Holzer *et al.* 2006; Rochette *et al.* 2009; Pescher *et al.* 2011). The study provided by Pescher *et al.* (2011) revealed important differences in intracellular survival and animal infection of axenic *L. donovani* amastigotes compared with host-derived ones. Axenic amastigotes differed in cell size and other attributes like expression of the A2 protein representing amastigote-specific marker (Charest and Matlashewski, 1994) responsible for protection of *L. donovani* from a variety of stresses (McCall and Matlashewski, 2012). Axenic amastigotes were less resistant to nutritional stress compared with splenic-derived ones, which resulted in strong attenuation in establishment of hamster infection (Pescher *et al.* 2011).

Importantly the transmission efficiency in our experiments was comparable for both modes of infection (42 and 56% in promastigote and amastigote initiated infections, respectively). Previous experiments with *L. infantum* suggest that the transmission rate is to a large degree influenced by the parasite species or even strain. Using the same methodologies Maia *et al.* (2011) demonstrated that 58% of *Phlebotomus perniciosus* and 65% of *L. longipalpis* transmitted *L. infantum* CUK3 strain, while only 33% of *P. perniciosus* and 14% of *L. longipalpis* transmitted *L. infantum* IMT373 strain. Additionally, the number of transmitted parasites has been reported as highly variable among individual females in several *Leishmania*/vector combinations; females typically transmit tens or hundreds of leishmania parasites but some individuals can deliver several thousand parasites (Warburg & Schlein, 1986; Kimblin *et al.* 2008; Maia *et al.* 2011). In the current study, however, all the females transmitted <600 parasites, we did not find individuals transmitting exceptionally high parasite load.

We conclude that regardless of the early decline in abundance of parasites in amastigote initiated infections and initial differences in representation of morphological forms, the *L. donovani* development ultimately results in equivalent pattern of infections in both experimental groups. Following defecation by female sand flies we did not find any significant differences in either course of infections or in the representation of metacyclics. Most importantly, sand flies of both experimental groups transmitted equivalent numbers of parasites to mammalian hosts. In summary, using promastigote stages for experimental infections of sand flies does not significantly alter the final outcome of leishmanial development in the vector and can be recommended for this purpose as the most technically convenient and appropriate method. However, for studies concerning early stage infection and development in sand flies (e.g. resistance of *Leishmania* to proteolytic enzymes or their escape from the peritrophic matrix), intracellular amastigotes are recommended for initiation of infections.
